# Core-shell heterostructured multiwalled carbon nanotubes@reduced graphene oxide nanoribbons/chitosan, a robust nanobiocomposite for enzymatic biosensing of hydrogen peroxide and nitrite

**DOI:** 10.1038/s41598-017-12050-x

**Published:** 2017-09-19

**Authors:** Veerappan Mani, Mani Govindasamy, Shen-Ming Chen, Tse-Wei Chen, Annamalai Senthil Kumar, Sheng-Tung Huang

**Affiliations:** 1 Department of Chemical Engineering and Biotechnology, National Taipei University of Technology, No.1, Section 3, Chung-Hsiao East Road, Taipei, 106 Taiwan (ROC); 2 Graduate Institute of Biomedical and Biochemical Engineering, National Taipei University of Technology, Taipei, Taiwan (ROC); 3 Nano and Bioelectrochemistry Research Laboratory, Department of Chemistry, School of Advanced Sciences, Vellore Institute of Technology University, Vellore, 632014 India

## Abstract

A robust nanobiocomposite based on core-shell heterostructured multiwalled carbon nanotubes@reduced graphene oxide nanoribbons (MWCNTs@rGONRs)/chitosan (CHIT) was described for the fabrication of sensitive, selective, reproducible and durable biosensor for hydrogen peroxide (H_2_O_2_) and nitrite (NO_2_^−^). The excellent physicochemical properties of MWCNTs@rGONRs such as, presence of abundant oxygen functionalities, higher area-normalized edge-plane structures and chemically active sites in combination with excellent biocompatibility of CHIT resulting in the versatile immobilization matrix for myoglobin (Mb). The most attractive property of MWCNTs@rGONRs which distinguishes it from other members of graphene family is its rich edge density and edge defects that are highly beneficial for constructing enzymatic biosensors. The direct electron transfer characteristics such as, redox properties, amount of immobilized active Mb, electron transfer efficiency and durability were studied. Being as good immobilization matrix, MWCNTs@rGONRs/CHIT is also an excellent signal amplifier which helped in achieving low detection limits to quantify H_2_O_2_ (1 nM) and NO_2_^−^ (10 nM). The practical feasibility of the biosensor was successfully validated in contact lens cleaning solution and meat sample.

## Introduction

Meeting the demand for cost-effective, robust and portable analytical devices, enzymatic biosensors have enormous potential as useful sensing tools in medicine, biofuel cells, food control, and in biomedical analysis^1,2^. The major step which influences the performance of a electrochemical enzymatic biosensor is ‘enzyme immobilization’ which refers to the proper coupling of enzymes to solid electrode to facilitate electronic communication between the electrode surface and prosthetic group of enzymes that deeply buried inside the protein backbone^3,4^. An ideal immobilization matrix should encompass characteristics like large surface area, good electrical conductivity, biocompatibility, high stability, good adhesion, inertness, affordability, physical strength, regenerability, ability to increase enzyme specificity/activity and hinder product inhibition and nonspecific adsorption^1^. Natural polymers, synthetic polymers, and inorganic materials are the traditional materials developed initially for enzymatic biosensors; however they are poor conductors^5^. Nanocomposites composed of carbonaceous nanomaterials (multiwalled carbon nanotubes (MWCNTs), graphene oxide (GO), graphene etc.,) and biopolymers are good immobilization matrix and they can enhance the signal sensitivity as well attributed to their outstanding electronic properties^6,7^. Therefore, carbonaceous nanomaterials made significant contribution in the development of highly sensitive biosensors^8,9^.

The core-shell heterostructured multiwalled carbon nanotubes@reduced graphene oxide nanoribbons (MWCNTs@rGONRs), a narrow strips of GO nanosheets can be prepared through longitudinal unzipping of MWCNTs and possess good physicochemical properties which includes large surface area, high conductivity, presence of abundant oxygen functionalities, available sites for covalent and non-covalent (π stacking) interactions^10^, good biocompatibility, chemical stability, and excellent mechanical, and thermal properties^11–13^. The most fascinating property of MWCNTs@rGONRs which distinguishes it from other members of carbon family is its high edge density and rich edge defects. In fact, the defect density of MWCNTs@rGONRs is higher than that of graphene or GO^14^. Since the electrochemical reactivity at edge planes is several orders of magnitude higher than that at basal planes, the rich edge defects of MWCNTs@rGONRs may leads to fast electron transfer process which has significant impact on the performance of biosensors. In addition, the way of MWCNTs@rGONRs preparation via longitudinal unzipping of MWCNTs creates plethora of structural defects which adds additional sites for immobilizing enzymes. Besides, the residual functional groups located at the edges of GONRs can facilitate the adsorption of analytes by π–π stacking, electrostatic, hydrogen bonding, and covalent interactions^15^. Recently, Au nanoparticles MWCNTs@rGONRs were successfully employed in the development of label-free impedimetric DNA biosensor and aptasensor for genetically modified soybean^16^ and acetamiprid^17^, respectively. GONRs based field effect transistor (FET) nanoelectronics was established for biosensing methylene blue^18^ and adenosine triphosphate molecule^19^. MWCNTs@rGONRs was used for the covalent immobilization of acetyl cholinesterase in order to develop Carbaryl biosensor^20^. Recently, Qian *et al*., revealed that MWCNTs@rGONRs possess intrinsic peroxidase-like catalytic activity^21^. More recently, Mehmeti *et al*., studied the wiring of apo-enzyme of glucose oxidase with graphene nanoribbons bound FAD to develop a bioelectrode for third generation glucose biosensor^22^.

Chitosan, a natural-biopolymer renowned matrix for enzyme immobilization attributed to its excellent biocompatibility, ability to form stable film, nontoxicity, biodegradability, high mechanical strength and hydrophilicity^23^. Since CHIT is a positively charged polymer in solutions, it can easily get adhered with negatively charged surfaces of MWCNTs@rGONRs^24^. Mb, an important heme containing redox enzyme has good biosensing property towards H_2_O_2_ and NO_2_^−^^9^. In the present work, we described a biocompatible nanobiocomposite based on chitosan (CHIT) encapsulated MWCNTs@rGONRs for the immobilization of myoglobin (Mb) and developed a sensitive biosensor for hydrogen peroxide (H_2_O_2_) and nitrite (NO_2_^−^).

The development of highly sensitive and robust biosensor device is important for the determination of H_2_O_2_ since H_2_O_2_ is now widely used as bleaching agent and disinfectant in pharmaceutical, medicinal, cosmeceutical and household cleaning products owing to its excellent antiseptic and anti-bacterial properties^25–27^. NO_2_^−^ in conjugation with salt has been widely used as food preservative in curing meats; however its excess level in blood causes formation of carcinogenic N-nitrosamine and hence the sensitive determination of NO_2_^−^ in meat samples is vital^28,29^. Being as good immobilization matrix for Mb, our study revealed that MWCNTs@rGONRs/CHIT is an excellent signal amplifier for the detections of H_2_O_2_ and NO_2_^−^. The main objective of this work is to develop a nanobiocomposite for Mb immobilization and a sensitive, reproducible, selective, and durable biosensor for H_2_O_2_ and NO_2_^−^.

## Results and Discussions

### Characterization of the material

**Figure 1:**
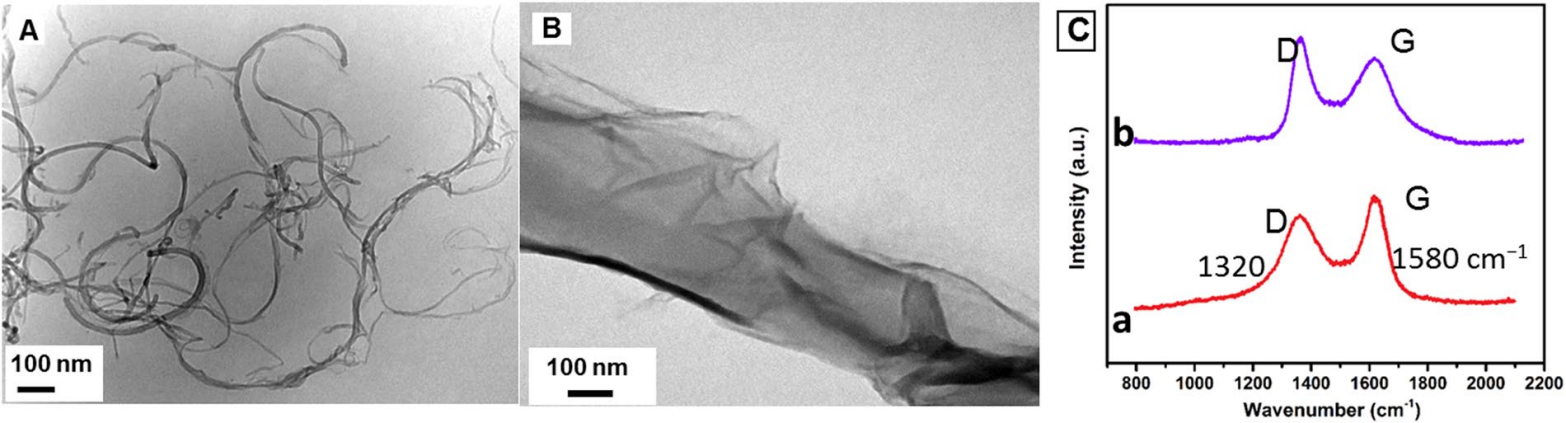
TEM images of MWCNTs (**A**) and MWCNTs@rGONRs (**B**). (**C**) Raman spectra of MWCNTs (a) and MWCNTs@rGONRs (b).

The TEM of MWCNTs (Fig. 1A) shows characteristic tubular image. The TEM of MWCNTs@rGONRs displayed ribbon-like morphology that is originated from unzipped outer walls of nanotubes (Fig. 1B) and this morphology is consistent with previous reports^21^. The Raman spectra of MWCNTs (curve a) and MWCNTs@rGONRs (curve b) illustrate two sharp peaks identified as D and G bands (Figure 1C)^15^. The intensity ratio of D to G band (I_D_/I_G_) has been significantly increased from 0.79 (MWCNTs) to 1.10 (MWCNTs@rGONRs). The significant increase in I_D_/I_G_ is attributed to the generation of numerous edge sites on the ribbons that caused decrease in average size of in-plane sp^2^ domain.

### Characterization of MWCNTs@rGONRs/CHIT/Mb and direct electron transfer of Mb

**Figure 2:**
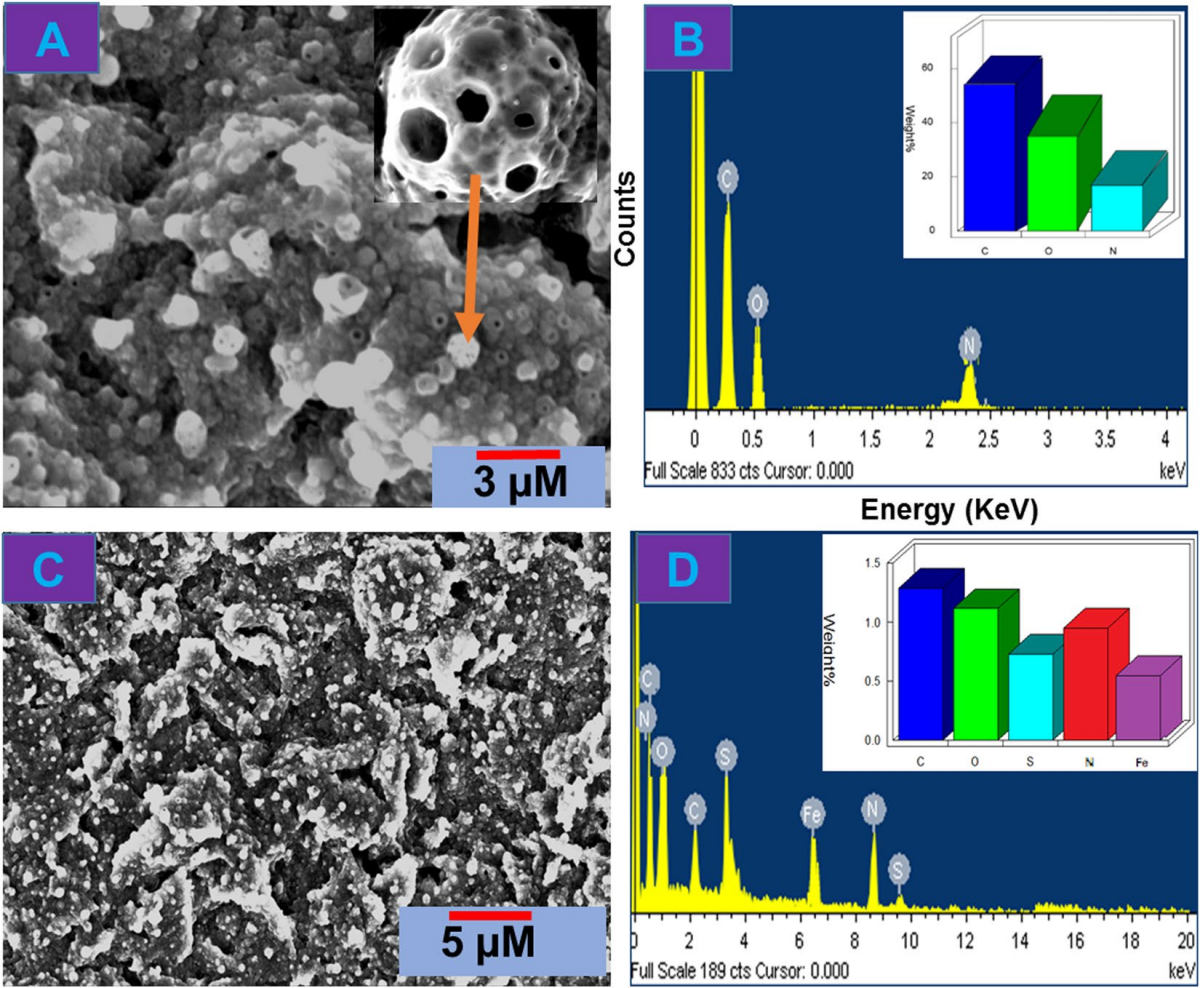
SEM images of MWCNTs@rGONRs/CHIT (**A**) and MWCNTs@rGONRs/CHIT/Mb (**C**). EDX spectra of MWCNTs@rGONRs/CHIT (**B**) and MWCNTs@rGONRs/CHIT/Mb (**D**).

The SEM image of MWCNTs@rGONRs/CHIT displayed highly porous and roughened network of chitosan entrapped GONR (Fig. 2A). The corresponding EDX spectrum of MWCNTs@rGONRs/CHIT (Fig. 2B) presented the signals for C, O and N atoms and their quantitative results were given as inset to Fig. 2B. The SEM image of MWCNTs@rGONRs/CHIT/Mb (Fig. 2C) displayed the Mb coverage over the surface of nanobiocomposite. The abundant oxygen functionalities and edge defects might provide additional accommodation sites which enabled high Mb loading. The EDX spectrum of the nanobiocomposite displayed signals for C, O, N, S and Fe (Fig. 2D). Here, the Fe and S signals were originated from Mb.

**Figure 3:**
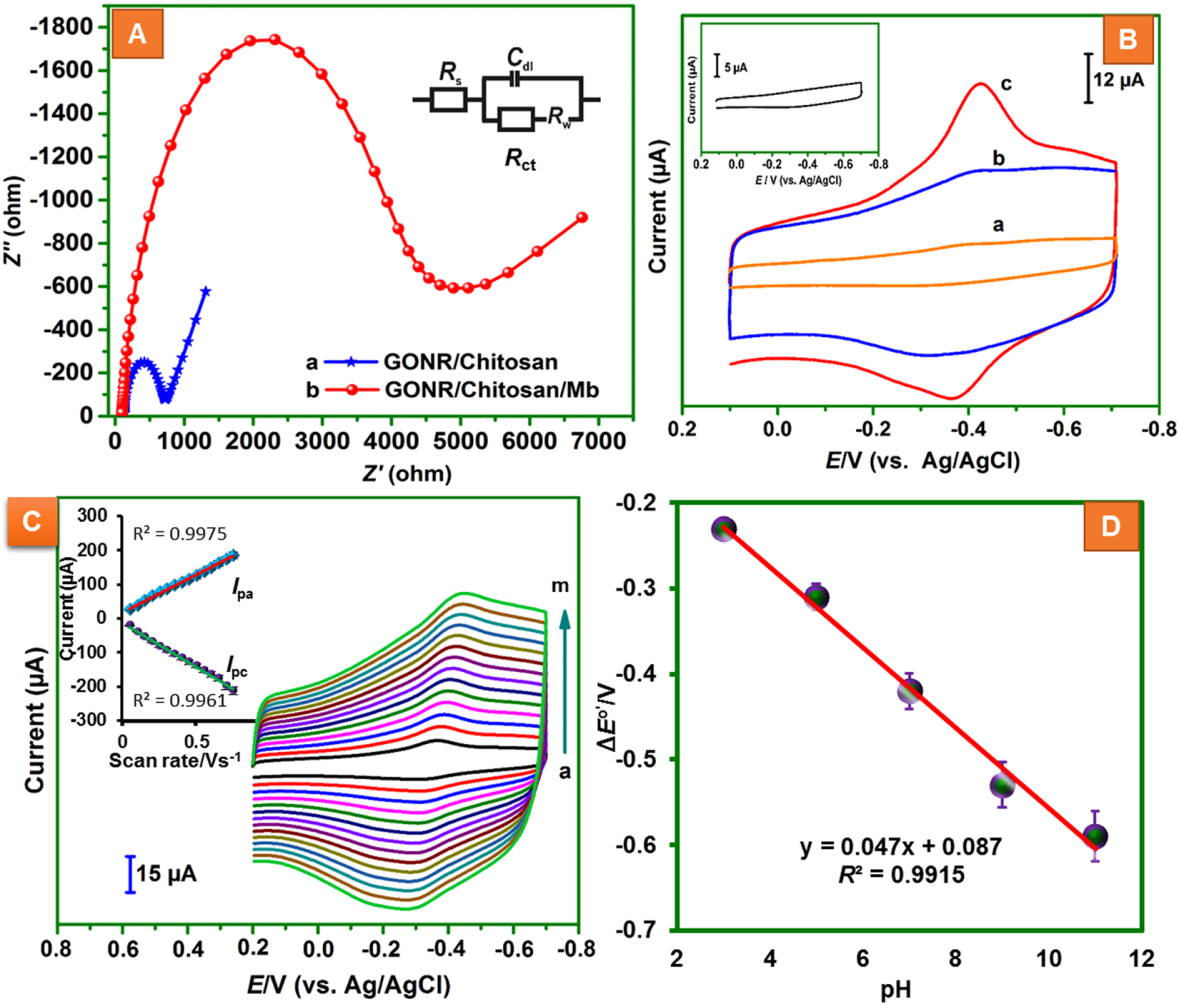
(**A**) EIS curves of GCE/MWCNTs@rGONRs/CHIT (a) and GCE/MWCNTs@rGONRs/CHIT/Mb (b) obtaiend in 0.1 M KCl containing 5 mM Fe(CN)_6_^3−/4−^. Amplitude: 5 mV, Frequency: 0.1 Hz to 100 kHz. Inset: Randles equivalent circuit; *R*_s_, *R*_ct_, *C*_dl_, and *W* are electrolyte resistance, charge transfer resistance, double layer capacitance and Warburg impedance, respectively. (**B**) CVs obtained at GCE/Mb (inset), GCE/CHIT/Mb (a), MWCNTs@rGONRs/Mb (b), and MWCNTs@rGONRs/CHIT/Mb (c) films modified GCEs in phosphate buffer (pH 7). Scan rate = 50 mVs^−1^. (**C**) Effect of scan rate: CV obtained at GCE/MWCNTs@rGONRs/CHIT/Mb in phosphate buffer (pH 7.0) at different scan rates (a = 0.05, b = 0.10, c = 0.15, d = 0.20, e = 0.25, f = 0.30, g = 0.35, h = 0.45, i = 0.50, j = 0.55, k = 0.60, l = 0.65, m = 0.70 and n = 0.75 V s^−1^); Inset: Plot of peak currents (µA) vs. scan rate (Vs^−1^). (**D**) Plot between formal potential (Δ*E* ^0^’) vs. pH; CVs were carried out at different pH.

Figure 3A displayed the EIS curves obtained for GCE/MWCNTs@rGONRs/CHIT (a) and GCE/MWCNTs@rGONRs/CHIT/Mb (b) in 0.1 M KCl containing 5 mM Fe(CN)_6_^3−/4−^. Randles equivalent circuit model (inset to Fig. 3A) was used to fit the experimental data (Here, *R*_s_, *R*_ct_, *C*_dl_, and *Z*_w_ are electrolyte resistance, charge transfer resistance, double layer capacitance and Warburg impedance, respectively). EIS measurements were represented as Nyquist plots. The *R*_ct_ values of GCE/MWCNTs@rGONRs/CHIT and GCE/MWCNTs@rGONRs/CHIT/Mb were 692 Ω and 4380 Ω, respectively. As expected, the value of *R*_ct_ was significantly increased after the immobilization of Mb, which is accounted for the increased resistance as a result of thick protein layers surrounding the FAD center of Mb^7^. Thus, EIS study revealed the successful immobilization of Mb on MWCNTs@rGONRs/CHIT

Direct electron transfer of Mb at nanobiocomposite was investigated by cyclic voltammetry in deoxygenated phosphate buffer (pH 7.0) (Fig. 3B). The potential range was 0.10 V to −0.70 V and the scan rate was 50 mV s^−1^. The cyclic voltammogram (CV) of GCE/MWCNTs@rGONRs/CHIT/Mb (c) exhibited a pair of well-defined quasi-reversible redox peaks with peak-to-peak separation (∆*E*_p_) of 35 mV at formal potential (*E* ^0^’) of −0.405 V. The redox peaks are correlated to the electron transfer of Mb which originated from the heme center Fe(III)/Fe(II)^9,30^. The highly enhanced peak currents indicated the attainment of direct electron transfer between electrode surface and heme center of enzyme and thus MWCNTs@rGONRs/CHIT is a verstaile matrix for Mb wiring to the solid electrode surface. About 92.5% of the initial redox peak currents were retained after 100 successive voltammogram cycles in phosphate buffer indicating good stability of the film due to the strong adhesion ability of the nanobiocomposite. However, the control electrodes (GCE/Mb (inset), GCE/CHIT/Mb (a) and GCE/MWCNTs@rGONRs/Mb (b)) have shown poor direct electron transfer ability as revealed by feeble redox peaks. In other words, the MWCNTs@rGONRs/CHIT composite provides better immobilization environment for Mb over control electrodes might be due to the proper combination of MWCNTs@rGONRs and CHIT that led to offer large surface area, additional porosity, extra sites for Mb accommodation and improved biocompatibility. The plethora of structural defects and residual functional groups located at the edges of GONRs can facilitate the Mb immobilization through all possible interactions such as, π–π stacking, electrostatic, hydrogen bonding, and covalent interactions.

Figure 3C displays the CVs obtained at MWCNTs@rGONRs/CHIT/Mb nanobiocomposite film modified GCE in phosphate buffer (pH 7.0) at different scan rates. The redox peak currents were linearly increased as the scan rate increased. The good linearity between peak currents and the scan rates is indicating surface-controlled redox reaction of Mb. The linear regression equations for the dependence of anodic peak current (*I*_pa_) and cathodic peak current (*I*_pc_) with respect to scan rates can be expressed as, *I*_pa_/μA = 221.23 *ν*/V + 17.25; *R*² = 0.998; *I*_pc_/μA = −257.9 *ν*/V−12.89; *R*² = 0.996; where, *ν*(Vs^−1^) is the scan rate (inset to Fig. 3C). The amount of active Mb on the modified electrode surface (*Г*) was calculated by substituting the slope of peak currents versus scan rate in the equation, *I*_p_ = *n* ^2^ *F* ^2^ *νAΓ*/4*RT* (where, *n* is the number of electrons transferred and *A* (cm^2^) is the electrode surface area and the constants *R*, *T* and *F* stands for their usual meanings). The value of *Γ* was obtained to be 9.7 × 10^−10^ mol cm^−2^ which is higher than the theoretical monolayer coverage, 1.89 × 10^−11^ mol cm^−2^ and GO/nafion composite (*Γ* = 7.68 × 10^−11^ mol cm^−2^)^31^. The apparent heterogeneous electron transfer rate constant (*k*_s_) for Mb at the modified electrode was calculated to be 1.96 s^−1^ using the Laviron equation, Log *K*_s_ = *α* log (1 − *α*) + (1 − *α*)log*α* − log(*RT*/*nFν*) − *α*(1 − *α*)*nF*∆*E*_p_/2.3*RT*; nΔ*E*_p_ > 0.20 V. (*α* is the charge transfer coefficient (~0.5) and the other parameters stands for their usual meanings). The *k*_s_ value of 1.96 s^−1^ is significantly higher than that obtained at the previously reported immobilization matrices, such as, graphene–Pt nanocomposite (0.584 s^−1^)^32^, Ag nanoparticles doped CNTs (0.41 s^−1^)^33^, nafion/MWCNTs (0.332 s^−1^)^34^ and hemoglobin/CHIT-MWCNTs/Au particles membrane (0.74 s^−1^)^35^. Thus, the MWCNTs@rGONRs/CHIT nanobiocomposite is an excellent mediator to shuttle the electrons quickly between the reactive sites of Mb and the electrode surface which can be manifested to the sp^2^ domain of MWCNTs@rGONRs. Also, the presence of oxygen functionalities are partially contributed to the electron transfer based reaction mechanisms. In order to determine durability of the electrode, GCE/MWCNTs@rGONRs/CHIT electrode was stored in phosphate buffer at 4 °C and the redox behaviour was monitored every day. The electrode retained 91.4% of its initial redox peak currents after its continuous usage of 3 weeks which indicating good storage stability of the electrode. The effect of pH on immobilized Mb was studied at different pH (from 3 to 11) and the value of *E*°’ showed good linearity with pH of the electrolyte (Fig. 3D). The linear regression equation was obtained as, *E*°’/V = −0.047 pH/(V/pH) + 0.087/V; *R*² = 0.991. The slope value (0.047 V/pH) is close to the theoretical value of 0.0576 mV/pH for a reversible reaction that involves with equal numbers of electrons and protons^9^.

### H_2_O_2_ biosensing at MWCNTs@rGONRs/CHIT/Mb film modified electrode

**Figure 4:**
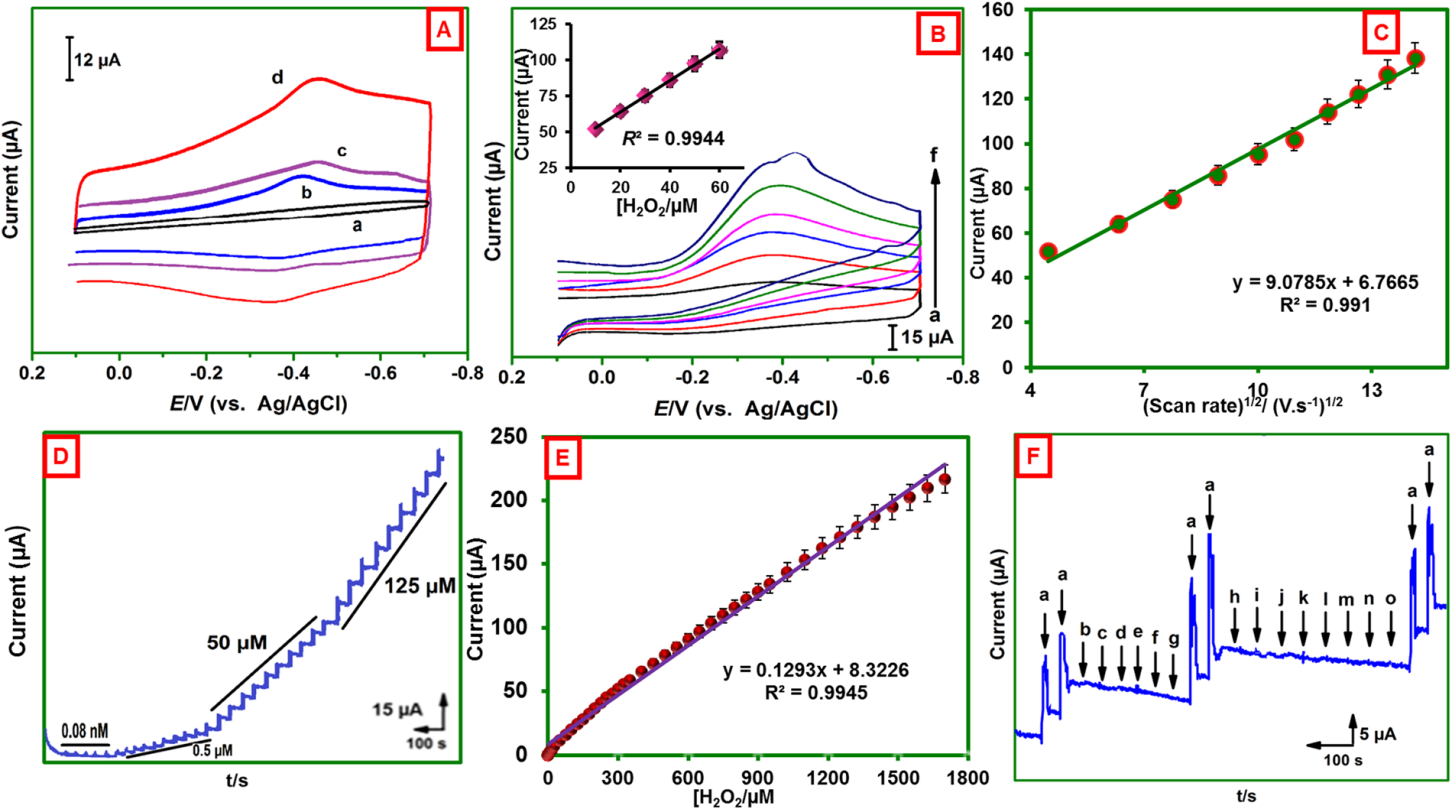
(**A**) CVs obtained at CHIT/Mb/GCE (a), MWCNTs@rGONRs/Mb/GCE (b), MWCNTs/CHIT/Mb/GCE (c), and MWCNTs@rGONRs/CHIT/Mb/GCE (d) in phosphate buffer (pH 7) containing 10 µM H_2_O_2_. Scan rate = 50 mV s^−1^. (**B**) CVs of GCE/MWCNTs@rGONRs/CHIT/Mb in phosphate buffer (pH 7.0) containing different concentrations of H_2_O_2_ (a = 10, b = 20, c = 30, d = 40, e = 50 and f = 60 µM). Inset: [peak current] vs. [H_2_O_2_]/µM. (**C**) [scan rate]^1/2^/(V.s^−1^)^1/2^ vs. [current]/µA. CVs obtained in phosphate buffer (pH 7.0) containing 10 µM H_2_O_2_ at different scan rates. (**D**) Amperometric response of GCE/MWCNTs@rGONRs/CHIT/Mb for different concentrations of H_2_O_2_. Rotation speed = 1200 rpm; *E*_app_ = −0.35 V. (**E**) calibration plot: [H_2_O_2_] (µM) vs. response current (µA). (**F**) Amperometric response of MWCNTs@rGONRs/CHIT/Mb for 10 µM H_2_O_2_ (a) and other species (10 µM of dopamine (b), uric acid (c), ascorbic acid (d), NADH (e), folic acid (f), cysteine (g), epinephrine (h), guanine (i), pyridoxine (j), cholesterol (k), caffeine (l), methionine (m), glucose (n) and nitrate (o)).

The electrocatalytic ability of the fabricated bioelectrode was employed to towards electrochemical reduction of H_2_O_2_. Cyclic voltammograms were performed at the potential range of 0.10 V to −0.70 V with scan rate of 50 mV s^−1^ (Fig. 4A). The electrocatalytic abilities of these electrodes are in following order: GCE/MWCNTs@rGONRs/CHIT/Mb > GCE/MWCNTs/CHIT/Mb > GCE/MWCNTs@rGONRs/Mb > GCE/CHIT/Mb. The electrodes (GCE/CHIT/Mb, GCE/MWCNTs@rGONRs/Mb, and GCE/MWCNTs/CHIT/Mb) have shown poor electrocatalytic ability. The GCE/MWCNTs@rGONRs/CHIT/Mb displayed sharp cathodic peak for H_2_O_2_ reduction at the potential of −0.35 V and the peak current is considerably higher than that of control electrodes. Remarkably, the H_2_O_2_ reduction ability of MWCNTs@rGONRs/CHIT/Mb is higher than MWCNTs/CHIT/Mb which indicating that the unzipping of MWCNTs has significant improvement towards electrocatalysis. In addition, the cathodic peak current was linearly increased as the concentration of H_2_O_2_ increased (Fig. 4B). The plot between cathodic peak currents and [H_2_O_2_] exhibited good linearity (inset to Fig. 4B). The possible interactions are electrostatic interaction between structural and edge defects of MWCNTs@rGONRs and H_2_O_2_. The presence of larger amount of edge plane-like defects and sp^2^ domains collectively accelerates the catalysis. Moreover, the surface enriched oxygen functionalities could significantly promoted the electrocatalysis^14^. The effect of scan rate on the reduction of H_2_O_2_ was investigated. The cathodic peak current was increased as the scan rate increased and the plot between peak current and square root of scan rate exhibited good linearity indicating diffusion controlled process (Fig. 4C).

Figure 4D displayed the amperometric responses obtained at MWCNTs@rGONRs/CHIT/Mb modified rotating disc electrode upon sequential additions of H_2_O_2_. The rotation speed of the electrode was 1200 RPM and the applied potential was (*E*_app_) −0.35 V. Aliquots of H_2_O_2_ were injected into the buffer at regular intervals of 50 s. Well-defined and sharp increments in the peak currents were clearly observed at each spiking. The response currents are linear with the concentration of H_2_O_2_ (Fig. 4E). The dependence of response currents with concentration of H_2_O_2_ was obtained as, *I*_p_/μA = 0.1293 [H_2_O_2_]/ μAµM^−1^ + 8.323; *R* ^2^ = 0.995. The linear range was 1 nM–1625 µM, the limit of detection (LOD) was 1 nM and the sensitivity was 0.616 µAµM^−1^cm^−2^. As revealed by the Table 1, the sensor performance of the MWCNTs@rGONRs/CHIT/Mb is either superior or comparable to the previously reported H_2_O_2_ sensors. The selectivity of the sensor was examined in order to test the interefernce of likely intereferences in H_2_O_2_ assay. Figure 4F displayed the amperometric responses of the modified electrode for the successive additions of 10 μM of H_2_O_2_ (a), dopamine (b), uric acid (c), ascorbic acid (d), NADH (e), folic acid (f), cysteine (g), epinephrine (h), guanine (i), pyridoxine (j), cholesterol (k), caffeine (l), methionine (m), glucose (n) and nitrate (o). The electrode delivered sharp signals for the H_2_O_2_ addition, but it does not responded to the addition of any other compounds indicating good selectivity of the electrode. 10 µM H_2_O_2_ was spiked into the buffer coexisted with the aforementioned compounds and the response current of the resulting solution was consisitent irrespecive of the associated compounds which indicating good specificity of the electrode. Therefore, the MWCNTs@rGONRs/CHIT/Mb electrode can be used for highly sensitive and selective determination of H_2_O_2_.

**Table 1:** Comparison of electroanalytical parameters of H_2_O_2_ obtained at MWCNTs@rGONRs/CHIT/Mb bioelectrode with previously reported electrodes

H_2_O_2_				NO_2_^−^			
Electrodes	LOD	Linear range/µM	Ref.	Electrodes	LOD	Linear range/µM	Ref.
Carbon nanodots–^a^CHIT	0.27	1–118	36	Pd/RGO	0.23	1–1000	37
MWCNTs/cysteamine/nafion	0.01	0.1–70.0	38	MWCNT/cysteamine/Nafion	0.1	1–250	38
Ag nanoparticles/Mb	0.088	1–3000	39	^d^CRGO	1	8.9–167	28
^b^MoS_2_/graphene/CNTs	0.83	5–145	26	RGO–MWCNT–Pt	0.93	1–12000	9
Ag/CNTs–^c^RGO	0.9	100–10^5^	40	Fe/graphene/MWCNT	75.6	0.1–1680	29
MWCNTs@rGONRs/CHIT/Mb	0.001	0.001–1625	This work	MWCNTs@rGONRs/CHIT/Mb	0.01	0.01–1350	This work

^a^CHIT = Chitosan, ^b^MoS_2_ = Molybdenum disulfide, ^c^RGO = Reduced graphene oxide, ^d^CRGO = Chemically reduced graphene oxide.

### Electrocatalysis and biosensing of NO_2_^−^

**Figure 5:**
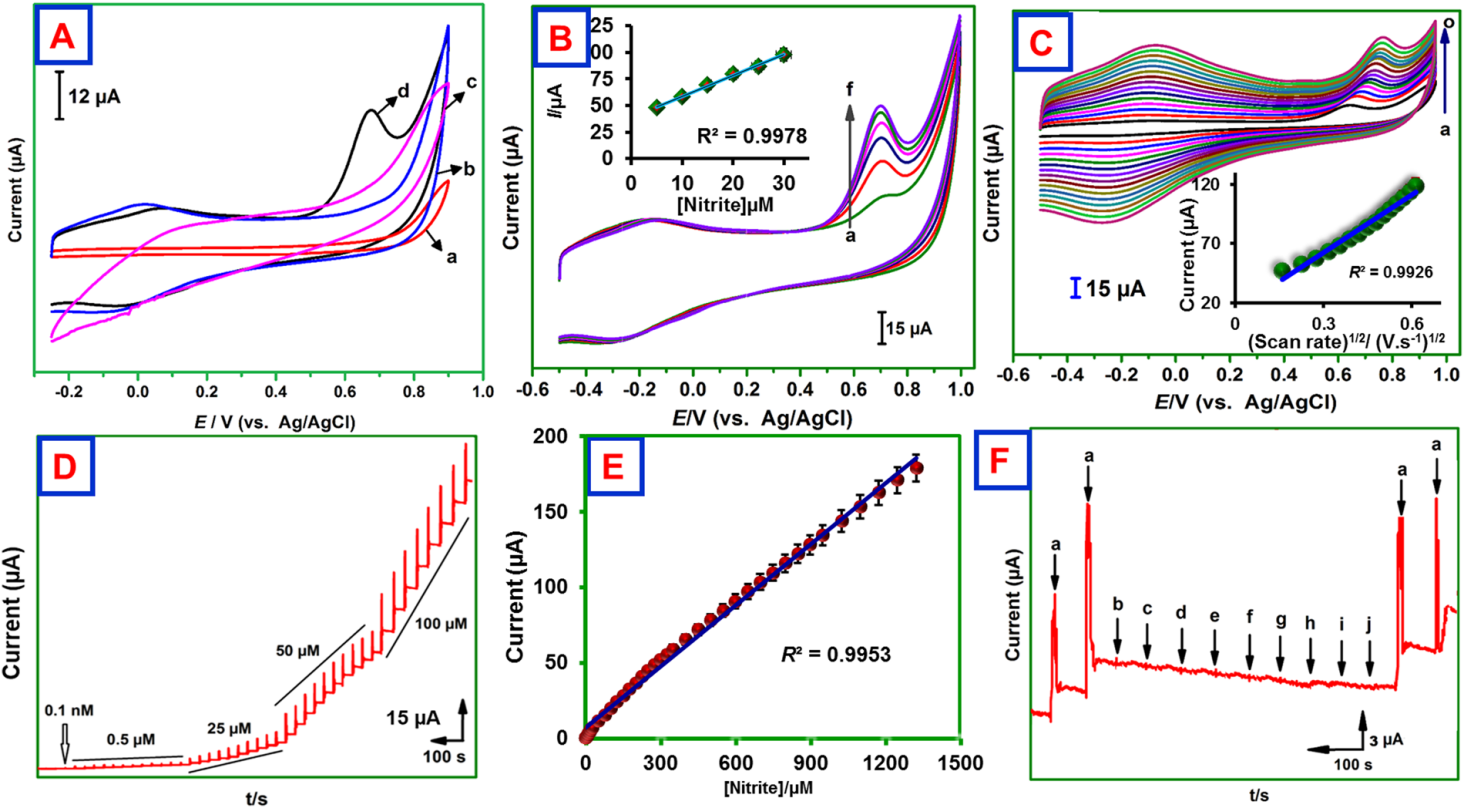
(**A**) CVs obtained at GCE/CHIT/Mb (a), GCE/MWCNTs@rGONRs/Mb (b), GCE/MWCNTs/CHIT/Mb (c) and GCE/MWCNTs@rGONRs/CHIT/Mb (d) in phosphate buffer (pH 7) containing 5 µM NO_2_^−^. Scan rate = 50 mV s^−1^. (**B**) CVs obtained at MWCNTs@rGONRs/CHIT/Mb/GCE in phosphate buffer (pH 7.0) containing different concentrations of NO_2_^−^ (a = 5.0, b = 10.0, c = 15.0, d = 20.0 and e = 30.0 µM). (**C**) CVs obtained at GCE/MWCNTs@rGONRs/CHIT/Mb in phosphate buffer (pH 7.0) containing 5 µM NO_2_^−^ at different scan rates from (0.02 to 0.3 Vs^−1^ (a = 0.02, b = 0.04, c = 0.06, d = 0.08, e = 0.10, f = 0.12, g = 0.14, h = 0.16, i = 0.18, j = 0.20, k = 0.22, l = 0.24, m = 0.26, n = 0.28 V and o = 0.30 s^−1^). (**D**) Amperometric response of MWCNTs@rGONRs/CHIT/Mb electrode upon successive additions of NO_2_^−^ into phosphate buffer (pH 7.0). Rotation rate = 1200 rpm; *E*_app_ = +0.70 V. (**E**) Calibration plot of [nitrite] vs. response current. (**F**) The amperometric response of MWCNTs@rGONRs/CHIT/Mb electrode for 10 µM NO_2_^−^ (a) and 10 µM of NH_4_Cl (b), NaF (c), KCl (d), NaCl (e), glucose (f), dopamine (g), cysteine (h), NADH (i) and nitrate (j).

Figure 5A displays the voltammograms obtained at GCE/CHIT/Mb (a), GCE/MWCNTs@rGONRs/Mb (b), GCE/MWCNTs/CHIT/Mb (c) and GCE/MWCNTs@rGONRs/CHIT/Mb (d). The scan rate was 50 mVs^−1^ and the potential was applied between −0.50 and 1.0 V. A well-defined and sharp irreversible anodic peak was obtained for the oxidation of NO_2_^−^ at +0.70 V at GCE/MWCNTs@rGONRs/CHIT/Mb indicated the efficiency of the modified electrode in promoting oxidation of NO_2_^−^. On the other hand, control electrodes (GCE/CHIT/Mb, GCE/MWCNTs@rGONRs and GCE/MWCNTs/CHIT/Mb) have shown poor electrocatalytic ability. Besides, the overpotential required to oxidize NO_2_^−^ is considerably lowered than the previously reported electrodes^9,28,29^. The peak current was linearly increased as the concentration of NO_2_^−^ (Fig. 5B). The effect of scan rate revealed that the oxidation of NO_2_^−^ follows diffusion controlled kinetics (Fig. 5C)^41^.

Figure 5D, displayed the amperogram acquired at MWCNTs@rGONRs/CHIT/Mb electrode upon each successive additions of NO_2_^−^. The *E*_app_ was +0.70 V. The plot between concentration of NO_2_^−^ and current exhibited good linearity and the respective linear regression equation was obtained as, *I*_p_/μA = 0.135 [NO_2_^−^] (μAμM^−1^) + 6.88; *R* ^2^ = 0.995 (Fig. 5E). The linear range was 10 nM–1350 µM. The LOD and sensitivity were calculated to be 10 nM and 0.643 µAµM^−1^ cm^−2^. The sensor performance was comparable to the previous reports (Table 1 **)**. As shown in the Table 1, the described sensor has shown lowest detection over many of the existing nitrite sensors. Selectivity of the electrode to detect NO_2_^−^ was tested in presence of likely interferences (NH_4_Cl (b), NaF (c), KCl (d), NaCl (e), glucose (f), dopamine (g), cysteine (h), NADH (i) and nitrate (j)). As shown in Fig. 5F, the modified electrode selectively and specifically detected NO_2_^−^ in the presence of other species indicating good selectivity of the electrode.

### Durability and reproducibility of the biosensor

The durability of the modified electrode was tested by monitoring the catalytic peak currents (−0.35 for H_2_O_2_ and 0.70 V for NO_2_^−^) every day. The sensor was stored at 4 °C when not in use. About 93.6% and 94.5% of the initial catalytic response was retained after 10 days of electrode’s continuous usage revealing good durability. This can be related to the good biocompatibility of the electrode which provides excellent microenvironment to the Mb; as a result the bioactivity was retained for many days. For the reproducibility test, CVs were recorded in phosphate buffer (pH 7.0) towards H_2_O_2_ and NO_2_^−^; The RSD values for five individual measurements were 4.62% and 4.25%, respectively demonstrating good reproducibility.

### Real sample analysis

**Figure 6:**
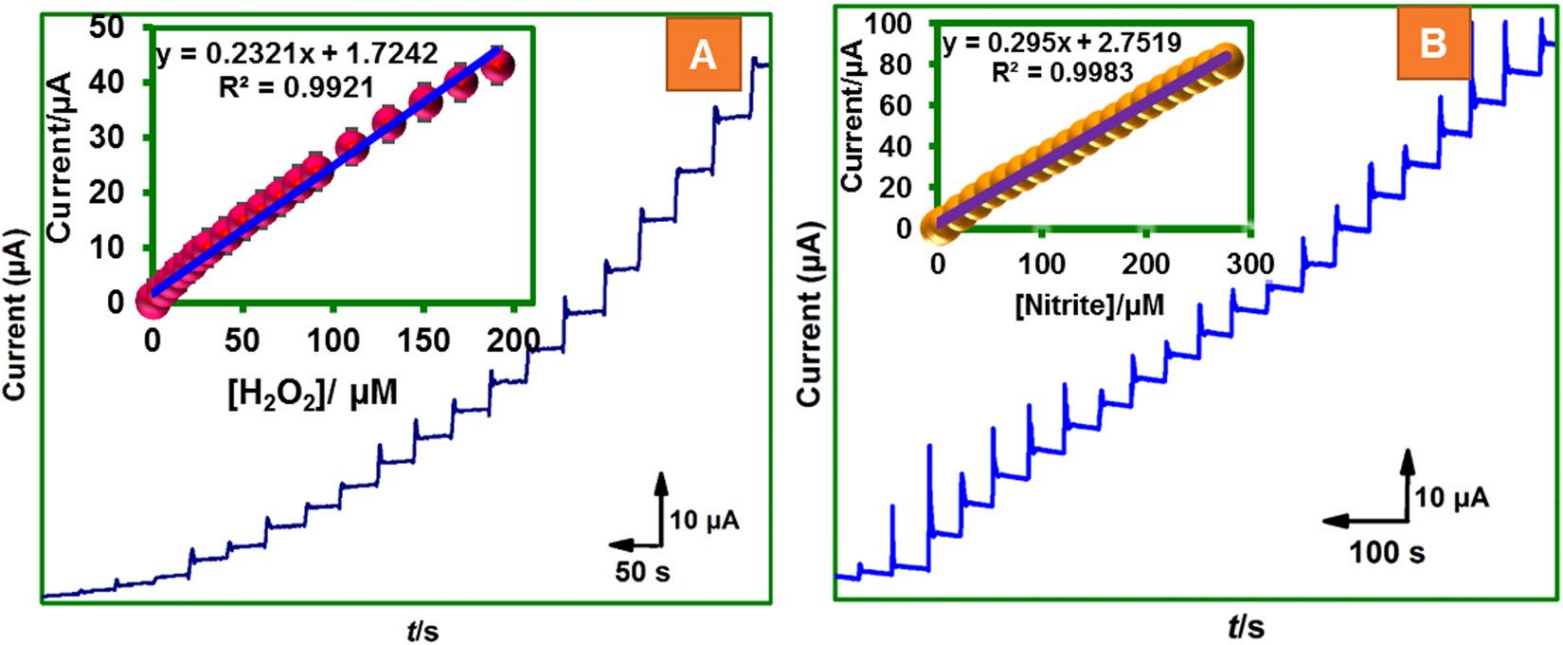
Amperometric response of MWCNTs@rGONRs/CHIT/Mb film modified electrode towards real samples. (**A**) H_2_O_2_ detection in contact lens solution (*E*_app_ = −0.35 V) and corresponding calibration plot (inset). (**B**) NO_2_^−^ sensing in beef sample (*E*_app_ = +0.70 V) and corresponding calibration plot (inset).

The practical feasibility of the fabricated biosensor was demonstrated in commercially available contact lens cleaning solution. The contact lens solution contains (3% H_2_O_2_) was directly tested via amperometry using our electrode and optimized amperometry procedure of lab sample. As shown in Fig. 6A, the electrode delivered prompt signals for each spiking of contact lens solution revealing the excellent sensing ability of the sensor. The response current was linear over 5 µM to 180 µM with detection limit of 1 µM (inset to Fig. 6A). In order to perform real sample analysis of NO_2_^−^ in beef sample, first beef sample (NO_2_^−^ free) was immersed in phosphate buffer and stirred for 20 min. Then the beef pieces were removed and the washed solution was spiked with known concentration of NO_2_^−^ and amperometry was performed by following optimized lab samples procedure. As shown in Fig. 6B, the sensor has shown good sensing performance as revealed by sensitive and prompt signals and the linear range was 6 µM to 275 µM with detection limit of 3 µM (inset to Fig. 6B). Thus, the biosensor has good practical feasibility and the developed biosensing platform can be applicable in the quantification of H_2_O_2_ and NO_2_^−^ in pharmacy and meat samples, respectively.

## Conclusions

A facile nanobiocomposite, MWCNTs@rGONRs/CHIT was demonstrated for the immobilization of Mb. The studies revealed that high amount of active Mb can be immobilized on the nanobiocomposite and the electron transfer rate between Mb and electrode surface was considerably higher than the previous reports. The bioelectrode was highly stable, biocompatible and durable. A highly sensitive, selective, reproducible and durable biosensor was fabricated for the determination of H_2_O_2_ and NO_2_^−^. The practical applicability of the biosensor was successfully validated in contact lens cleaning solution and meat sample. The developed nanobiocomposite can be applicable for the immobilization of other enzymes as well. The nanobiocomposite can be a potential matrix for the immobilization of other enzymes or biomolecules as well.

## Experimental

### Materials and Instrumentation

Mb, MWCNTs (bundled > 95%, O.D × I.D × length of 7–15 nm × 3–6 nm × 0.5–200 μm) were purchased from sigma-Aldrich and used as received. All the reagents used were of analytical grade and used without any further purification. Contact lens cleaning solution containing 3% H_2_O_2_ was purchased from local pharmacy. Beef samples were purchased from local supermarket. The supporting electrolyte used for the electrochemical studies was 0.1 M phosphate buffer solution, prepared using Na_2_HPO_4_ and NaH_2_PO_4_ and the pH was adjusted either using H_2_SO_4_ or NaOH. Prior to each experiment, the electrolyte solutions were deoxygenated with pre-purified nitrogen gas for 10 min unless otherwise specified.

The electrochemical measurements were performed using CHI 611 A work station. The electrochemical studies were carried out in a conventional three electrode cell using BAS GCE as a working electrode (area 0.071 cm^2^), saturated Ag/AgCl as a reference electrode and Pt wire as a counter electrode. Amperometric measurements were performed with analytical rotator AFMSRX (PINE instruments, USA) with a rotating disc electrode (RDE) having working area of 0.24 cm^2^. Scanning electron microscopy (SEM) studies were performed using Hitachi S-3000 H scanning electron microscope. Energy-dispersive X-ray (EDX) spectra were recorded using HORIBA EMAX X-ACT (Sensor + 24 V = 16 W, resolution at 5.9 keV). EIM6ex Zahner (Kronach, Germany) was used for electrochemical impedance spectroscopy (EIS) studies.

### Fabrication of MWCNTs@rGONRs/CHIT/Mb nanobiocomposite

MWCNTs@rGONRs was prepared by previously reported procedure with little modification^12^. Briefly, 250 mg of MWCNTs were added to 75 mL H_2_SO_4_ and stirred for 1 h. Then, 7 mL H_3_PO_4_ was added and the solution was continued to stir for another 20 min. Next, 2.5 g of KMnO_4_ was added and the whole solution mixture was heated at 65 °C for 2 h and finally cooled to room temperature. Finally, the reaction mixture was poured onto 100 mL of ice containing 10 mL 30% H_2_O_2_. A brown colored sediment was formed which was filtered and washed with 100 mL of water. Next, it was washed with 3 × with HCl (20 vol%, 30 mL each), 2 × with ethanol (30 mL each) and 2 × with ether (30 mL each). The purified MWCNTs@GONRs slurry was vacuum dried for overnight at 60 °C and redispersed in water to get 1 mg mL^−1^.

MWCNTs@GONRs (1 mg) was dispersed in 1 mL of 0.25 wt.% CHIT solution through ultrasonication for 30 min. 6 µL suspensions of MWCNTs@GONRs/CHIT was drop-casted on the pre-cleaned GCE surface and dried at ambient conditions. Subsequently, the electrode was transferred to an electrochemical cell containing deoxygenated phosphate buffer (pH 5.0) and 15 consecutive cycles of cyclic voltammograms were performed at the potential range between 0 and −1.5 V^42^. The electrochemical reduction was performed in order to partially restore electrical conductivity. Next, the GCE/MWCNTs@rGONRs was washed with water and 6 µL dispersion of Mb (10 mg mL^−1^ in phosphate buffer) was drop casted and the electrode was allowed to dry at room temperature for 1 h. Finally, the MWCNTs@rGONRs/CHIT/Mb nanobiocomposite film modified electrode (bioelectrode) was gently washed with water to remove loosely adsorbed Mb. As control, MWCNTs@rGONRs/Mb and CHIT/Mb were prepared individually.
